# Lipid profile in healthy human volunteers before and after consuming ghee

**DOI:** 10.6026/97320630018742

**Published:** 2022-09-30

**Authors:** Kaarna Munisekhar, Sharan B Singh M, PVLN Srinivasa Rao, B Sitaram, N Sharvani, VS Kiranmayi, D Hemalatha

**Affiliations:** 1 Sri Padmavathi medical college, Tirupati, India; 2 Depatment of Dravyaguna,SriVenkateswaraAyurvedic College and Hospital, Tirupati, India; 3 Panchakarma, S.V Ayurvedic College and Hospital, Tirupati- 517501, India

**Keywords:** Cow ghee, prospective interventional study, lipid profile

## Abstract

Food is a cause of concern due to its effect on health and disease. Diet affects the occurrence and progress of non-communicable diseases, including hypertension, diabetes mellitus, cardiovascular diseases (CVD), and cancers. The exact dietary composition that helps in the prevention of diseases is not known. A higher intake of processed foods, sugar-sweetened beverages, Trans and saturated fats, and a lower intake of fresh fruits, vegetables, nuts, and whole grains are generally considered as a poor-quality diet. Therefore, it is of interest to document the lipid profile in healthy human volunteers before and after consuming ghee. Fasting serum lipids were measured before and after the intervention. The effect of the intervention on all the subjects was analysed by comparing the post-intervention data. Data shows that TC and LDL-C are significantly decreased. However, other parameters showed insignificant change. The effect of the intervention on the normolipidaemia group was also analysed. There was no significant change. Thus, data shows that cow ghee consumption is not harmful to health.

## Background:

Diet affects the occurrence and progress of non-communicable diseases, including hypertension, diabetes mellitus, cardiovascular diseases (CVD), and cancers [1]. A higher intake of processed foods, sugar-sweetened beverages, trans- and saturated fats, and a lower intake of fresh fruits, vegetables, nuts, and whole grains is generally considered as a poor quality diet [2]. Some dietary interventions that help prevent non-communicable diseases include decreased intake of saturated fatty acids (SFAs) and Tran's fatty acids (TFAs) and an increased intake of unsaturated fats [3]. Reduced consumption of fats has been one of the essential recommendations for the primary prevention of CVD [4]. The relationship between increased consumption of fats and higher serum cholesterol levels which increased the risk of atherosclerosis is known [5]. The type of dietary fat was also observed to play a prominent role in the development of CVD [6]. SFAs containing 12-16 carbon atoms increase low-density lipoprotein cholesterol (LDL-C), while stearic acid (C18:0) does not raise plasma cholesterol levels [7]. The effect of lipids on increasing plasma total cholesterol (TC) levels is known. TFA in hydrogenated oils are more harmful as they increase LDL-C, TC, and apolipoprotein B (Apo B) levels and decrease high-density cholesterol (HDL-C) and apolipoprotein A (Apo A) levels [8]. National health institutions of several countries have recommended a decreased intake of fats along with a reduction in the consumption of TFA [9]. Ghee lipids are not only rich in SFA but also contain cholesterol, which is oxidised while heating [10]. The incidence of heart disease among the Indian immigrant population is higher than that among other ethnic people [11]. Consumption of ghee and the regular Indian dietary regimen has been attributed to the increased risk of CVD in the Indian population [12]. Ghee is known to improve memory, develop immunity, lubricate the walls of GIT and facilitate easy digestion; hence is considered to induce many beneficial effects on human health [13]. Coronary atherosclerosis involving blood vessels of vital organs that leads to myocardial infarction and ischemia of the brain resulting in infarction is known [14]. Kumar et al. [15] showed the effects of feeding different levels of ghee ranging from 0.25 to 10% of total dietary fat for eight weeks on the serum and liver lipid levels of male Wistar rats when compared to rats fed with diets containing groundnut oil. Shankar et al. [16] showed the effect of ghee consumption on serum lipid profile. Mohammadifardn et al. [17] observed that subject who consumed ghee oil demonstrated a significant decrease in triglyceride and increase in Apo A levels. Zeb et al. [18] studied the effects of co-administration of unoxidized and thermally oxidized ghee in comparison to thermally oxidized ghee on lipid profile, haematological parameters, and liver tissue of rabbits. Therefore, it is of interest to document the lipid profile in healthy human volunteers before and after consuming ghee.

## Material and Methods:

### Study design:

The designed study is prospective interventional in nature.

### Place of study:

The present study was conducted in the Department of Physiology, Sri Venkateswara Institute of Medical Sciences (SVIMS), Sri Padmavathi Medical College for Women Tirupati, in association with the Department of Biochemistry, SVIMS, and Sri Venkateswara Ayurvedic Hospital, Tirupati, Andhra Pradesh, India.

### Recruitment of subjects:

After obtaining regulatory clearance from the institutional ethics committee (IEC no. 913), registration of study (CTRI/2020/06/026556), and prior informed written consent, the subjects were requested to fill the Proforma to obtain past medical history and food habits by 24-hour recall questionnaire. Healthy subjects aged 18-60 years were selected from Sri Padmavathi Medical College (W), and Sri Venkateswara Ayurvedic Hospital Tirupati, Andhra Pradesh, India. Those fulfilling the inclusion and exclusion criteria and who are willing to supplement with cow ghee orally were recruited into the study.

### Inclusion and exclusion criteria:

Healthy individuals aged 18 to 60 years and those willing to participate in the study were recruited.Consumption of non-vegetarian diet, Current smokers, Chronic alcoholism, Hypertension, Diabetes mellitus, Thyroid disorders, Renal and liver diseases, coronary artery disease, acute and chronic inflammatory diseases, Lipid-lowering drugs, those on vitamins and antioxidants, Pregnant and lactating women, and those not willing to participate in the study were the exclusion criteria.

### Details of intervention:

Once the volunteers were recruited into the study, they were given 1,470 grams of native Ongole breed cow ghee (prepared by the investigator) for six weeks and advised to consume 35 grams everyday [19, 20]. The compliance was verified by telephonic confirmation on alternate days. The volunteers were advised to bring the empty bottle at the end of the supplementation. During this period, the subjects were requested to consume a relatively constant vegetarian diet, maintain a relatively constant level of physical activity, and make no changes in the cooking medium.

### Sample collection:

Five ml of venous blood was collected into plain tubes from all the subjects following an overnight fast. The blood sample was allowed to clot, and the serum was separated by centrifugation at 2000 revolutions per minute for 10 minutes. The separated serum was transferred into appropriately labelled and marked aliquots and stored at -80°C until further analysis.

### Biochemical analysis:

All the biochemical parameters were analysed using standard methods. The details of the method, kits, and equipment used are shown in Table 1(see PDF).

### Statistical analysis:

The distribution of data was checked by the Kolmogorov Smirnov test. All categorical variables were summarized as count and percentage. Continuous variables were expressed as mean ± SD. The pre-post comparison within each group was made by paired t-test or McNemar's test. A result with a p-value less than 0.05 will be considered statistically significant. All the statistical analysis will be performed by using Microsoft Excel spreadsheets and the Statistical package for social sciences (SPSS) for windows version 20.0.

## Results:

A total of 154 subjects willing to participate in the study were screened. Of these, 89 subjects were excluded. The reasons for exclusion were hypertension (15 subjects), diabetes mellitus (24 subjects), cardiovascular disease (5 subjects), thyroid disorders (10 subjects), non-vegetarians (22 subjects), and refused to participation (13 subjects). There were five dropouts due to COVID infection.

### Sample size calculation:

The initially proposed sample size based on other studies was 51. Sample size calculation with the obtained results USINGn Master VERSION 2.0 developed by CMC VELLORE with 80 % Power, Alpha Error of 5- and 2-sided variance was found to be adequate for total cholesterol and LDL cholesterol. However, the sample size was inadequate for other parameters studied. As the sample size was adequate for the primary objective and main some secondary objectives of the study, the sample size was considered adequate.

### Distribution of data:

Distribution of data of TC (mg/dL), TGL (mg/dL), HDL-C (mg/dL), VLDL-C (mg/dL) and LDL-C (mg/dL) was checked using Kolmogorov smrinov test and histograms.

### Data analysis:

Data analysis was done by including all subjects [n=60], subjects with normolipidaemia [n=37] and subjects with dyslipidaemia [n=23]. The distribution of the parameters studied in each group was studied using the Kolmogorov Smirnov test and histograms. The majority of the parameters studied did not show normal distribution in either pre-intervention or post-intervention or both in all the three groups studied. Hence, the significance of the change in parameters was tested using a non-parametric test, the Wilcoxon rank-sum test. The Chi-square test compared the number of subjects showing increase and decrease.

### Statistical analysis:

The distribution of data will be checked by the Kolmogorov Smirnov test. All categorical variables will be summarized as count and percentage. Continuous variables will be expressed as mean ± SD or median with the interquartile range depending on the distribution of data. The pre-post comparison within each group will be made by paired t-test or McNemar's test. A result with a p-value less than 0.05 will be considered statistically significant. All the statistical analysis will be performed by using Microsoft Excel spreadsheets and the Statistical package for social sciences (SPSS) for windows version 20.0.

## Discussion:

Food consumption, which largely depends on production and distribution, determines the health and nutritional status of the population [19]. Hypercholesterolemia, obesity, and oxidative stress are the major risk factors for developing atherosclerosis and cardiovascular diseases in humans [20-23]. However, numerous synthetic drugs are available for decreasing cholesterol levels. In the present prospective interventional study, the effect of desi cow ghee in the diet at 35 g /day for six weeks. The results indicate that ghee consumption of 35g per day up to 6 weeks contributes to the energy intake in the diet of healthy, young, physically active volunteers on a vegetarian diet. The effect of the intervention on all the 60 subjects studied. The TC, TG, HDL, VLDL, and LDL were decreased after the intervention of ghee. However, the LDL-C was statistically significant. In the normo-lipidemia group, 37 volunteers have studied. The lipid profile in the normo-lipidemia group shows a change in the expected direction but is not statistically significant. In the dyslipidemic group, 23 volunteers were studied. The TC and LDL-C are statistically significant. However, the dyslipidemia group's TG, HDL, and VLDL show a change in the expected direction but are not statistically significant. This data is similar to Sharma et al. [13] who compared the various lipid profile parameters among the three study groups when all the subjects were analyzed (n=200). Group C (ghee group) had significantly decreased TC, TG, VLDL, LDL, TC/HDL, and LDL/HDL compared to groups A and B. This is similar to Shankar et al. [16] where ghee has no significant effect on the serum lipid profile when consumed in less than 10% of total energy intake compared to mustard oil. Mohammadifard et al. [17] data from 206 healthy participants from Iran showed that ghee significantly reduced the TC and TGL compared to hydrogenated oil. Data from Nirmala et al. showed decreased TC, LDL, VLDL, and TGL levels and HDL levels were increased in all the groups, which was not statistically significant in the 5% cream and 5% curd ghee groups. Kumar et al. [15] has shown that the consumption of up to 10% ghee in the diet positively affected serum lipid profiles in Wistar rats. There was a dose-dependent decrease in TC, LDL, VLDL, and TGL when ghee was given at levels greater than 2.5% in the diet. Liver cholesterol and triglycerides were also decreased. The ghee was the sole source of fat at a 10% level; PUFA in the serum and liver lipids were significantly reduced. Kumar et al. [15] showed the effect of medicated ghee on serum lipid profile levels in psoriasis patients. They demonstrated the hypo-lipidemic effects of ghee when given at high doses. The Patients were given incremental doses of 60 mL of medicated ghee every day over seven days in their study. There was an 8.3% decrease in serum, TC a 26.6% decrease in TC in TGL, a 17.8% decrease in serum phospholipids, and a 15.8% decrease in serum cholesterol esters. Chinnadurai et al. [23] shows that high CLA enriched ghee increases the antioxidant enzyme activities. Feeding high CLA enriched ghee also leads to a decrease in TC, TGL, and HDL and, in turn, decreases the LDL-C, reducing the atherogenic index and proving its anti-atherogenic property. The frequency of CHD was less among the subjects who consumed more ghee and less oil per month [24]. The CHD was positively correlated with increased oil intake, the blood level of TG, TC, LDL, VLDL, TC/HDL, and LDL/HDL ratio.

The hypo-cholesterolemic actions of dairy products may be mediated by inhibiting cholesterol biosynthesis and enhancing the faecal excretion of sterols and bile acids. The ghee contains CLA, which has been shown to decrease serum LDL and atherogenesis in a rabbit model. Serum oleic acid levels increased when the animals were fed ghee-supplemented diets may enable LDL to resist oxidation, preventing plaque formation. In a follow-up study on the mechanism of hypo-cholesterolemic ghee, diets supplemented with 2.5 and 5% ghee, both native and oxidized ghee, were fed to Wistar rats [15]. The diets were prepared isocaloric with groundnut oil. Dietary ghee did not affect the HMG CoA reductase activity in liver microsomes, indicating that it did not affect cholesterol biosynthesis, significantly increased the excretion of bile constituents, and decreased serum cholesterol levels. The liver is the primary site for cholesterol biosynthesis, regulated by HMG CoA reductase. This enzyme is down regulated by cholesterol concentration in the diet and inhibited by oxysterols. Even though the heated ghee significantly decreased TC by 10-25% in the serum and 7-14% in the intestinal mucosal cells compared to the control group fed groundnut oil. There was a corresponding decrease in cholesterol ester fractions in the serum and intestinal mucosa, indicating that ghee lipids inhibited the esterification process in the intestine. Cholesterol excretion in the bile of these animals was significantly increased by 18 - 30%. Bile is an essential mode of transport for cholesterol excretion and its metabolites. There was a significant increase in phospholipids' excretion, total bile acids, and uronic acid. The authors concluded that cow ghee exhibited hypo-cholesterolemic effects by increasing the secretion of biliary constituents. For thousands of years, ghee has been heavily utilized in Ayurveda for its health-promoting properties. It is administered alone and used with herbs to treat various disorders. There are 55 to 60 types of medicated ghee described in Ayurvedic texts [25]. Positive results have been reported in research conducted on several mixtures containing ghee. In the last two to three decades, ghee has been implicated in the increasing prevalence of CAD in Asian Indians living outside India and upper socioeconomic classes living in towns and cities in India [26]. The data available in the literature do not support a conclusion about the harmful effects of the moderate consumption of ghee in the general population. Moreover, Asian Indians previously had a low incidence of CAD and used ghee in their cooking for generations, which is low in PUFAs such as linoleic acid and arachidonic acid. The epidemic of CAD in India began two to three decades ago when traditional fats were replaced by oils rich in linoleic and arachidonic acid [26]. 40% of vanaspati contains trans-fatty acids [27]. It should be noted that adulteration of commercially prepared ghee with vanaspati is also prevalent in India [28].

## Conclusions:

Data shows the effect of inclusion of desi cow ghee in diet at 35g/day for six weeks for predisposition of atherosclerosis. Inclusion of desi cow ghee in the diet at 35g/day for six weeks does not produce dyslipidaemia, i.e., an increase in TC/TGL/LDL/VLDL or decrease in HDL in normo-lipidaemic subjects. It improves dyslipidaemia, i.e., a decrease in TC/LDL. Thus, ghee consumption is not harmful to health. However, long-term studies, including a larger population, may be required before advocating this in the dietary regime.
